# Trivariate Empirical Mode Decomposition via Convex Optimization for Rolling Bearing Condition Identification

**DOI:** 10.3390/s18072325

**Published:** 2018-07-18

**Authors:** Yong Lv, Houzhuang Zhang, Cancan Yi

**Affiliations:** 1 Key Laboratory of Metallurgical Equipment and Control Technology, Wuhan University of Science and Technology, Ministry of Education, Wuhan 430081, China; lvyong@wust.edu.cn (Y.L.); houzhuangwust@163.com (H.Z.); 2 Hubei Key Laboratory of Mechanical Transmission and Manufacturing Engineering, Wuhan University of Science and Technology, Wuhan 430081, China

**Keywords:** trivariate empirical mode decomposition, convex optimization, low-rank matrix approximation, rolling bearing condition identification

## Abstract

As a multichannel signal processing method based on data-driven, multivariate empirical mode decomposition (MEMD) has attracted much attention due to its potential ability in self-adaption and multi-scale decomposition for multivariate data. Commonly, the uniform projection scheme on a hypersphere is used to estimate the local mean. However, the unbalanced data distribution in high-dimensional space often conflicts with the uniform samples and its performance is sensitive to the noise components. Considering the common fact that the vibration signal is generated by three sensors located in different measuring positions in the domain of the structural health monitoring for the key equipment, thus a novel trivariate empirical mode decomposition via convex optimization was proposed for rolling bearing condition identification in this paper. For the trivariate data matrix, the low-rank matrix approximation via convex optimization was firstly conducted to achieve the denoising. It is worthy to note that the non-convex penalty function as a regularization term is introduced to enhance the performance. Moreover, the non-uniform sample scheme was determined by applying singular value decomposition (SVD) to the obtained low-rank trivariate data and then the approach used in conventional MEMD algorithm was employed to estimate the local mean. Numerical examples of synthetic defined by the fault model and real data generated by the fault rolling bearing on the experimental bench are provided to demonstrate the fruitful applications of the proposed method.

## 1. Introduction

With the development of industrial production, the key equipment and structure always have the characteristics of complex structure, variable operation status and continuous online service. Especially in the metallurgical industry, most parts such as rolling bearing are working under an environment of high speed, overloading and high temperatures. As an important approach of structural health monitoring, the damage feature extraction algorithm for rolling bearings based on signal processing technology has important significance [1,2,3]. Naturally, the vibration signal is expressed as nonlinear, non-stationary and time-varying [4,5,6,7,8]. Moreover, the noise components directly affect the performance of the signal analysis [9,10,11]. Thus, improved signal processing methods are required.

In order to improve the signals to noise ratio (SNR) of actual measured vibration signal, many scholars have done a lot of research work [12,13,14,15,16]. Short-time Fourier transform (STFT) is presented as a modified edition of conventional Fourier transform, which is used to analysis non-stationary signals [17]. Since the size and shape of analysis window are fixed, it cannot achieve adaptability with the change of the target signal. Based on it, Wavelet Transform (WT) is proposed by the inner product operator between the analysis signal and the mother wavelet function [18,19]. Moreover, the multi-scale analysis is conducted by choosing different factors. The performance is still related to the basis function and decomposition level selection. So far, the research has focused on two radically different techniques. The first set of techniques is known as reassignment methods, while the second set of algorithms is referred to as data driven algorithms. Synchronous wavelet transform (SWT) is regarded as the most common application reassignment method. It can produce highly localized time-frequency representations in the scale domain [20]. However, it may be unsuitable for fast varying instantaneous frequency. Empirical Mode Decomposition (EMD) is a typical signal adaptive decomposition algorithm. If the signal contains intermittent modes, its performance is affected by the mode-mixing problem (MMP) [21,22]. Thus, by adding random white noises, Ensemble EMD (EEMD) and its improved edition have been proposed [23,24,25]. In essence, the problem of MMP, namely the stability of decomposition, determines the ultimate performance of signal decomposition.

The above-mentioned signal processing method of time-frequency analysis is mainly oriented to the single sensor. The single sensor can only provide limited useful information, and the characteristics of the early equipment running status information are hard to be inspected. On the contrary, the multi-channel signals processing technology can be employed to identify the multichannel correlation, improve the accuracy and reliability of diagnosis. In short, it can provide more scientific evaluation to equipment online service quality [26]. Especially the sensor and measurement technology have made tremendous progress, multivariate signal processing method is the focus of this paper. Since EMD has presented a better potential, it can be set up as a powerful way in multi-channel signal processing. The concept of bivariate empirical mode decomposition (BEMD) was firstly proposed by Rilling [27]. By using the rotation property of quaternions, Empirical mode decomposition for trivariate signals (TEMD) is presented, which is employed to obtain multiple projections on the sphere [28]. Then, a multivariate extension of EMD has been proposed by Rehman, namely multivariate empirical mode decomposition (MEMD) [29]. As a critical step in signal decomposition, the uniformly sampled scheme is used to estimate the local mean envelope of BEMD and MEMD input signals in multiple directions in multi-dimensional space. In solving the problem of the decomposition stability, MEMD has more superiority than BEMD and conventional TEMD, it has been widely used in signal processing and other fields [30,31]. When the MEMD is used to identify the early damage status identification of the rolling bearing, the multi-channel data in high-dimensional space is usually inhomogeneous, and the feature information will be inferred by noise components. The noise components are generated by thermal shock, acoustic noise, base strain, the thermal noise inside the amplifier circuit and so on. It will be added to the original true data and produce measurement error. This phenomenon usually indicates that it is difficult to identify the real fault characteristic frequency information under strong noise environment. Therefore, the MEMD method has some shortcomings. For instance, it is sensitive to the noise components and has poor signal decomposition ability for complex signal. Generally, the vibration signal is measured by three sensors located in different positions and the trivariate signal is expressed as the form of matrix. Distinguished from MEMD, trivariate empirical mode decomposition based on low-rank matrix approximation via convex optimization framework and non-convex regularization term is proposed firstly to achieve better denoising performance. It has been proved that the non-convex penalty functions can obtain more accurate low-rank structure [32]. Then, considering the inhomogeneity of trivariate data distribution in high-dimensional, a non-uniformly sampled scheme based on singular value decomposition (SVD) is presented to obtain low-rank trivariate data matrix. The obtained principal component is corresponding to the directions of highest curvature, and the main features of the data can be sampled. Subsequently, a novel trivariate empirical mode decomposition is put forward in this paper for rolling bearing condition identification. In order to verify the validity of the method, the simulation signal model and measured experimental data of fault rolling bearing are conducted to analysis.

This paper was organized as follows: the basic principles and characteristics of the trivariate empirical mode decomposition via convex optimization were introduced in the second chapter. In the third section, the numerical simulation analysis was conducted to confirm the effectiveness of the algorithm. In the fourth part, the validity of the proposed method was verified by the bearing data of the experimental bench. The conclusions of the study were given in section fifth.

## 2. Theoretical Descriptions

Given a trivariate signal $Y(t)=\left\{y_{1}(t),y_{2}(t),y_{3}(t)\right\}$ generated by the mechanical equipment using triaxial acceleration sensor or three sensors installed in different positions, it is a common fact that the actual measured trivariate signal is composed by useful signal and noise components. The problem of estimating a low-rank matrix X from its noisy observation Y is considered as follows:

(1) $$Y=X+W\;Y,X,W\in {\mathbb{R}}^{3\times n}\;$$

where W represents zero-mean additive white Gaussian noise. 

It was reported that the non-convex regularization methods outperformed the convex regularization methods on matrix approximation and could enhance sparsity [32]. Thus, in this paper, a low-rank matrix approximation [33] for the trivariate signal Y can be expressed as the following model:

(2) $$\arg\;\underset{X}{\min}\{\psi (X)=\frac{1}{2}||Y-X|{|}_{F}^{2}+\lambda_{0}\sum_{i=1}^{k}\phi (\sigma_{i}(X);a_{0})+\lambda_{1}\sum_{i=1}^{m}\sum_{j=1}^{n}\phi (X_{ij};a_{1})\}\;$$

where k=min(m,n), ϕ is the parameterized non-convex penalty function, $\sigma_{i}(X)$ represents the singular values of the matrix X. $a_{0}$ and $a_{1}$ represent the parameters of non-convex penalty functions, which are associated with the convexity of the objective function. The weights of two parameterized non-convex penalty functions are adjusted by the regularization parameters $\lambda_{0}$ and $\lambda_{1}$. Obviously, if $\lambda_{1}=0$ and ϕ(x,a)=|x|, Equation (2) is transformed into a typical nuclear norm minimization problem. 

Typical non-convex penalty functions should be 2nd order differentiable, continuous and symmetric. An example of a non-convex penalty function is the rational penalty function, which is defined as:

(3) $$\phi (x,a)=\frac{\left|x\right|}{1+a\left|x\right|/2}\;$$

Equation (3) is parameterized by the parameter a≥10. The proximal operator of ϕ(x,a) is expressed as:

(4) $$prox_{\phi }(y,\lambda ,a)=\arg\underset{x\in R}{\min}\left\{\frac{1}{2}{\left(y-x\right)}^{2}+\lambda \phi (x,a)\right\}\;$$

It is noted that the proximity operator is associated with the non-convex function ϕ(x,a). In order to ensure that the objective function ψ(X) is strictly convex, the parameter $a_{0}$ and $a_{1}$ should meet the following requirement:

(5) $$0\leq a_{0}\lambda_{0}+a_{1}\lambda_{1}<1\;$$

Then, the alternating direction method of multipliers (ADMM) algorithm in conjunction with variable splitting is employed to solve the trivariate data matrix of the low-rank approximation problem. The ADMM algorithm is applied to Equation (2), and yields the following iterative procedure. Firstly, initialize the model with Z=0 and D=0. Then, the iterative optimization is conducted as following:

(6) $$X\leftarrow prox_{\phi }(\frac{1}{1+\mu }(Y+\mu (Z+D)),\frac{\lambda_{1}}{1+\mu },a_{1})\;$$

(7) [U,Σ,V]←SVD(X−D) 

(8) $$Z\leftarrow U\cdot prox_{\phi }(\Sigma ,{\lambda_{0}/\mu ,a}_{0})\cdot V^{T}\;$$

(9) D←D−(X−Z) 

where μ>1 is the scalar augmented Lagrangian parameter to ensure the strictly convex of the algorithm; SVD indicates the operator of singular value decomposition. The trivariate data preprocessing method based on low-rank matrix approximation via convex optimization requires the specification of two regularization parameters $\lambda_{0}$ and $\lambda_{1}$, two penalty parameters $a_{0}$ and $a_{1}$, and the scalar augmented Lagrangian parameter μ. Generally, the regularization parameters $\lambda_{0}$ and $\lambda_{1}$ are determined by the follow formulation:

(10) $$\lambda_{i}=\beta_{i}\sigma \;$$

where $\beta_{i}$ is chosen so as to maximize the signal-to-noise ratio. σ is corresponding to the spectral noise variance, which can be estimated by the wavelet coefficients by using the local variance analysis [34]. The values of two penalty parameters $a_{0}$ and $a_{1}$ can be simplified chosen by Equation (5). It’s also noted that the value of μ does not affect the solution due to the iterative algorithm converges, and μ=1.5 is always suitable for algorithm convergence. Through the processing of the proposed method, a low-rank approximation matrix X is obtained from the observed spectral data matrix X.

Then, the singular value decomposition (SVD) of the covariance matrix to X is performed, $E\left\{XX^{T}\right\}=V\Lambda V^{T}$, where V is the eigenvector matrix and $\Lambda =diag\left\{\lambda_{1},\lambda_{2},\lambda_{3}\right\}$ is the eigenvalues corresponding to the eigenvalue matrix. The corresponding azimuth angle $\phi_{H}$ and inclination angle $\theta_{H}$ of the Hammerseley projections are obtained by uniformly sampling a sphere using the Hammerseley sequence [29]. It is aimed to identify the Cartesian coordinates of the uniformly sampled sphere. Thus, the non-uniform sample $e_{p}$ on a sphere can be determined by constructing an ellipsoid with the following parameters:

(11) $$e_{l}=\left[\begin{array}{c} \lambda_{{}_{1}}^{\frac{1}{3}}\cos\theta_{H}\sin\phi_{H}\\ \lambda_{{}_{2}}^{\frac{1}{3}}\sin\theta_{H}\cos\phi_{H}\\ \lambda_{{}_{3}}^{\frac{1}{3}}\cos\phi_{H} \end{array}\right]\;$$

The direction of the highest curvature is sampled by rotating the ellipsoid $e_{l}$. Therefore, the novel non-uniform samples scheme can be defined as $e_{p}=Ve_{l}=\left\{e_{1},e_{2},e_{3}\right\}$. As a critical step of signal decomposition, the local mean estimation according to the conventional MEMD algorithm using the non-uniform samples $e_{p}$ is implemented and the computational procedure of trivariate empirical mode decomposition via convex optimization can be described in Algorithm 1.

**Algorithm 1.** Trivariate empirical mode decomposition via convex optimization
Perform the low-rank matrix approximation via convex optimization framework to a trivariate signal Y(t) and the new observed signal X(t) can be obtained.The projection $p^{k}(t)$(k=1,2,…,K) can be calculated of the input low-rank trivariate signal X(t) along the direction vector $\left\{e_{1}{}^{k},e_{2}{}^{k},e_{3}{}^{k}\right\}$. It should also be noted that K is the number of the directional vector sets.The time instants $t_{m}^{k}$ corresponding to the maxima of the set of projected signals $p^{k}(t)$ is determined.Interpolate [$t_{m}^{k}$, $X(t_{m}^{k})$] to obtain multivariate envelope curves $E^{k}(t)$. Then, the envelop mean can be calculated $M(t)=\frac{1}{K}\sum_{k=1}^{K}E^{k}(t)$.Calculate the residual by R(t)=X(t)−M(t). If the stopping criterion condition of iteration can be satisfied, then R(t) is set as one IMF and repeat the above steps to X(t)−R(t) until the next IMF is isolated. If it does not satisfy the stopping criterion, then repeat the above steps to R(t) until it meets the criterion.


Cauchy-type convergence condition is chosen as the stopping criterion of the sifting iterative process, which indicates the iterative will stop under the circumstance that the discrepancy between adjacent sifting results is less than a threshold with a range 0.2–0.3. For clarify, K=32 is always suitable for the application of trivariate signal empirical mode decomposition.

## 3. Simulation Signal Analysis

Rolling bearing is an important transmission component supporting the rotating part of the mechanical equipment. If a bearing with an outer race that is fixed to the structure, a typical fault rolling bearing model can be simplified as follows [35]:

(12) $$x_{i}(t)=\alpha \sin(2\pi f_{i}t)[1+\beta \sin(2\pi f_{r}t)]\;$$

where $f_{i}$ is the inner ring failure frequency and $f_{r}$ is corresponding to rotational frequency, respectively. Then, the following three source signals $X=\left\{x_{1},x_{2},x_{3}\right\}$ are employed to simulate the collected vibration signals in this section:

(13) $$x_{1}(t)=0.1\cos(2\pi f_{1}t+10)\;$$

(14) $$x_{2}(t)=0.2\sin(2\pi f_{2}t-15)\;$$

(15) $$x_{3}(t)=0.3\sin(2\pi f_{i}t)[1+\sin(2\pi f_{r}t)]\;$$

where the characteristics frequency about three simulation source signals are chosen as $f_{1}=10$ Hz, $f_{2}=45$ Hz, $f_{i}=110$ Hz, $f_{r}=10$ Hz. The sampling number and sampling frequency are set as N=1024 and $f_{s}=1024$ Hz, respectively. 

In the analog process of sensor collecting signal, the vibration signal will be collected by any sensor simultaneously, and the trivariate signal is the instantaneous mixed signal composed by three above mentioned simulation signal. Considering the influence of noise, the zero-mean additive white Gaussian noise with variance 0.5 is added to the observation signal, and it is denoted as $S_{N}$. A random matrix of 3 × 3 is chosen to mix the simulation source signal together and it can be expressed as follows:

(16) $$A=\left(\begin{array}{ccc} 0.6241 & 0.3674 & 0.8852\\ 0.6791 & 0.9880 & 0.9133\\ 0.3955 & 0.0377 & 0.7962 \end{array}\right)\;$$

**Figure 1:**
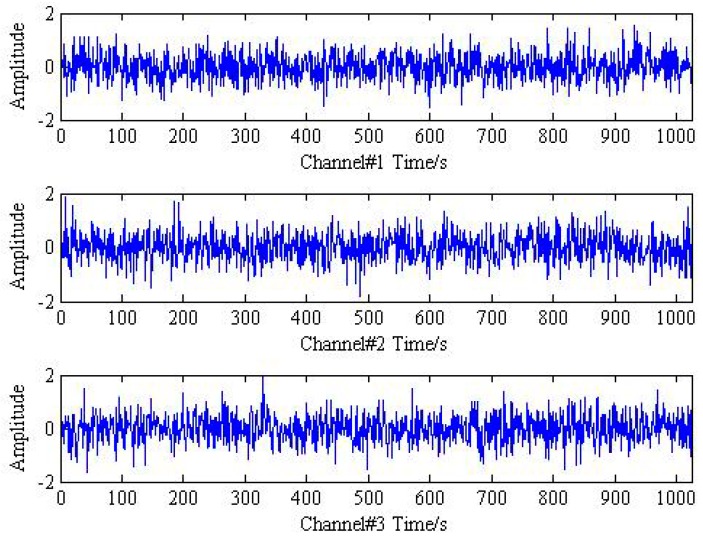
The time response of mixed trivariate signal with noise.

**Figure 2:**
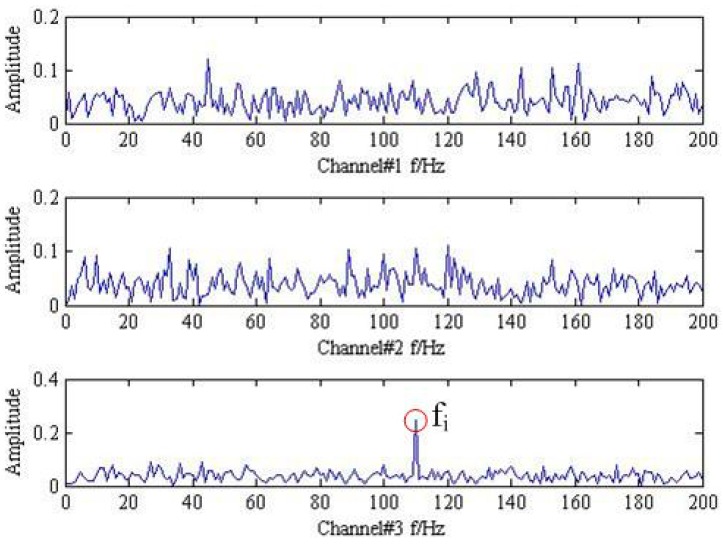
The frequency spectrum of mixed trivariate signal with noise.

Then, the instantaneous mixed signal model can be described as $Y=AX+S_{N}$ and $Y=\left\{y_{1},y_{2},y_{3}\right\}$, which indicates trivariate signal with noisy generated by three source signal using three sensors. The analysis results of trivariate signal in time-domain and frequency spectrum provided by FFT are shown in Figure 1 and Figure 2.

**Figure 3:**
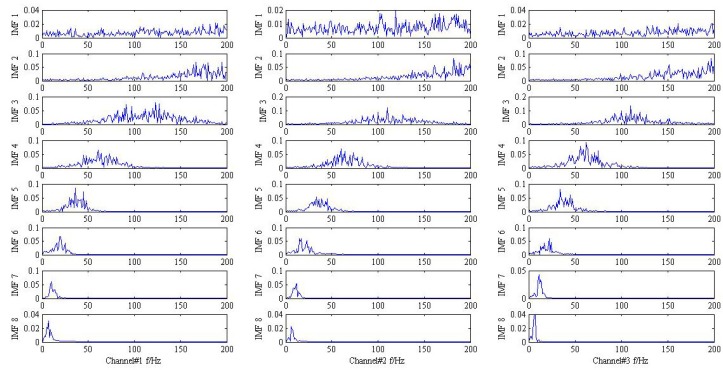
The result provided by the MEMD method.

As shown in Figure 1 and Figure 2, it is hard to identify the frequency characteristics and modulation phenomenon of the observed trivariate signals with noisy. Only the inner race fault frequency $f_{i}$ in channel#3 can be inspected, and other frequency components are not obvious for all the channels. It is illustrated that the influence of noise should not be neglected and more advanced methods are required. Then, the conventional MEMD methods are employed to deal with the simulation signal and the result is plotted in Figure 3. From the analysis result of the three channels shown in Figure 3, it is proved that MEMD has advantages in guaranteeing the stability of the decomposition results. However, the characteristic frequencies $f_{1}$, $f_{2}$ and the frequency modulation $f_{i}\pm f_{r}$ still cannot be identified.

**Figure 4:**
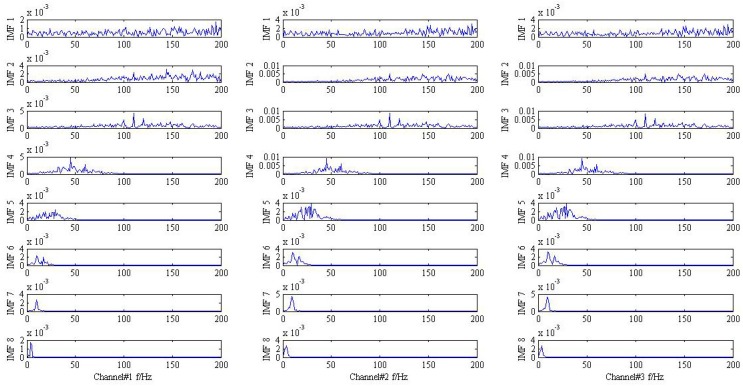
The result provided by the proposed method.

**Figure 5:**
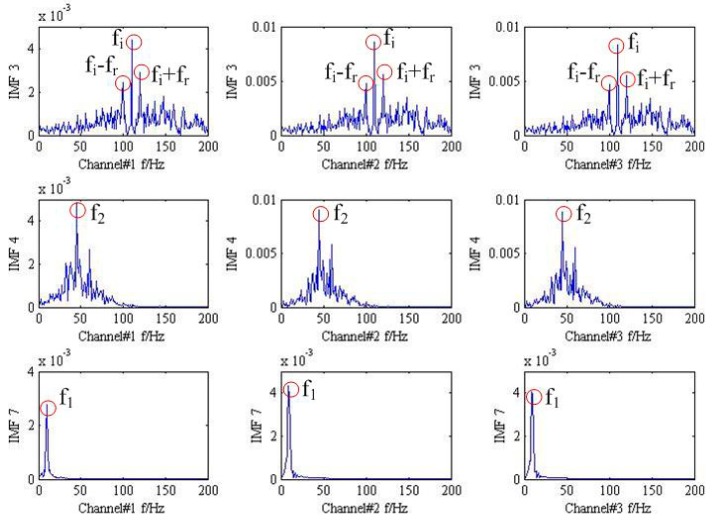
3th, 4th, and 7th IMFs of different signal in the frequency domain.

Eventually, the proposed method is applied to simulate trivariate signal and the result is drawn in Figure 4. For clarify the denosing performance, the 3th, 4th, and 7th IMFs are chosen according to the maximum similarity with the original signal shown in Figure 5. It is obvious that the frequency modulation $f_{i}\pm f_{r}$ can be found in the 3th IMF. In addition, the characteristic frequencies $f_{1}$, $f_{2}$ also can be viewed in the 4th and 7th IMF, respectively. The result provided by the proposed method is coincides with practical situation and the effective can be demonstrated.

## 4. Analysis of the Roll Bearing on the Experimental Bench

**Figure 6:**
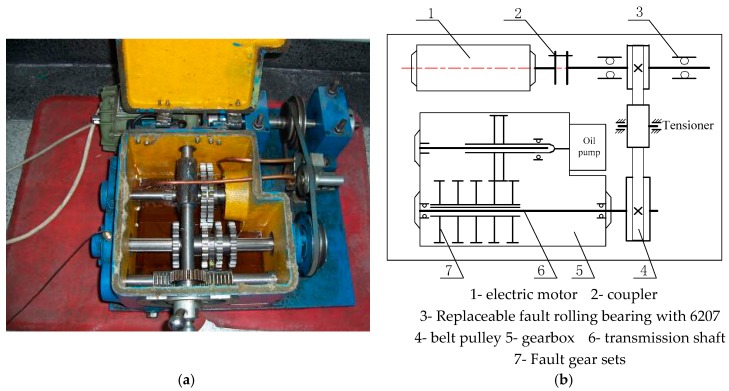
Fault experimental table of roll bearing. (**a**) The physical map of test rig; (**b**) The structure diagram of test rig.

Commonly, the measured vibration signal is more complicated than the simulation signal in the process of actual signal analysis. In order to verify the effectiveness of the proposed algorithm of trivariate empirical mode decomposition via convex optimization, the outer ring failure signal of rolling bearing in the experimental device is analyzed. The experimental apparatus and its structure diagram are drawn in Figure 6, where bench comprises a drive shaft which is driven by 550 W (220 V/50 Hz) AC motor. 

**Figure 7:**
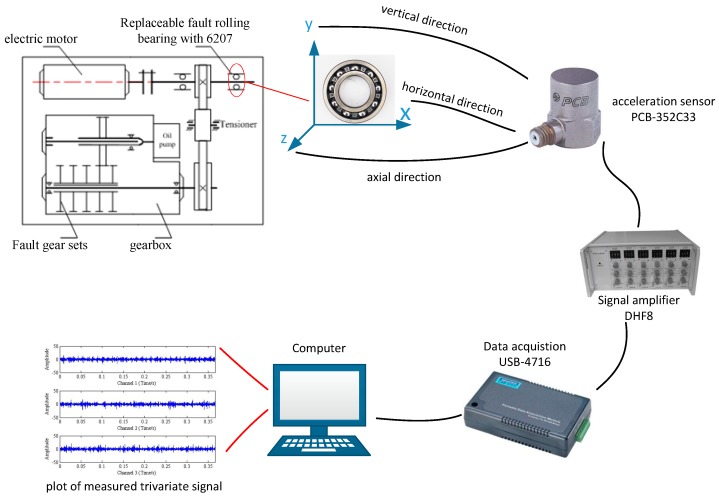
Testing flow chart for experimental bench.

The electric spark machining method is used to carry out pitting treatment on the outer ring of the replaceable bearing to simulate the outer faults. A PCB-352C33 acceleration sensor is used to collect the acceleration signals. The installation position of sensor is located in the horizontal direction, vertical direction and axial direction of the bearing on the right side of the experimental platform. The bearing type is deep groove ball bearing with model number of 6207. The external diameter of removable bearing was *D =* 72 mm and the inner diameter was *d =* 35 mm, respectively. The testing flow chart for experimental bench is demonstrated in Figure 7 and the sampling frequency is determined as 16,000 Hz. Vibration detection in horizontal (*x*), vertical (*y*) and axial (*z*) directions should be considered as far as possible, which is aimed to collect the trivariate signal. Suppose there is no relative sliding between the raceway surface and the rolling elements, and the outer ring is fixed. Then, the outer fault frequency 87.01 Hz can be obtained. The detailed experimental parameters are shown in Table 1.

**Figure 8:**
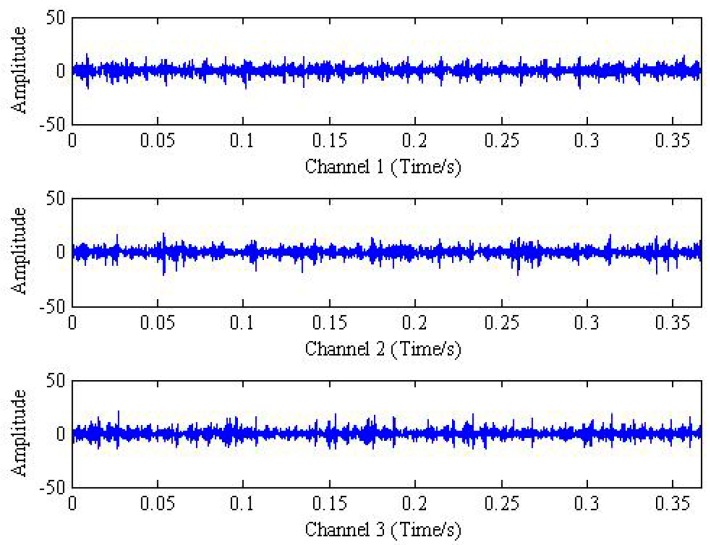
The time domain plot of measured trivariate signal.

**Figure 9:**
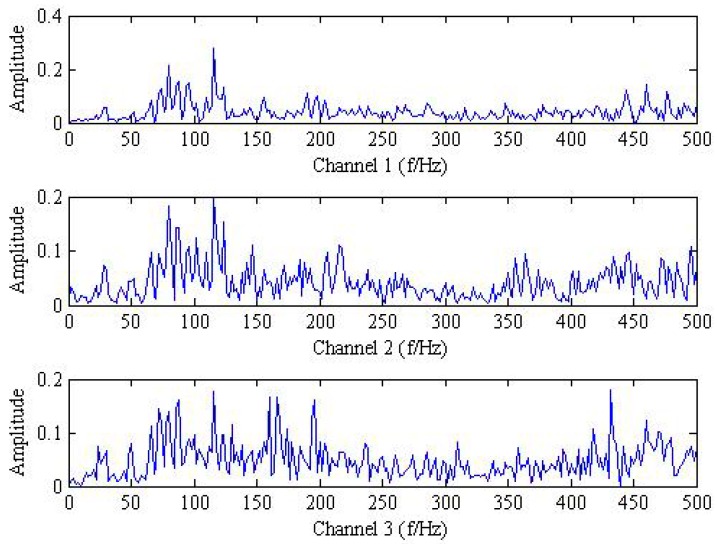
The frequency domain plot of measured trivariate signal.

It can be seen from Table 1 that the rotation frequency is $f_{r}=24.17$ HZ. According to the theory of bearing fault diagnosis, the frequency of outer ring fault signal of rolling bearing is calculated as $f_{o}=87.01$ HZ. Then, the time domain and frequency domain plots of the original measured vibration signal are shown in Figure 8 and Figure 9, respectively.

**Figure 10:**
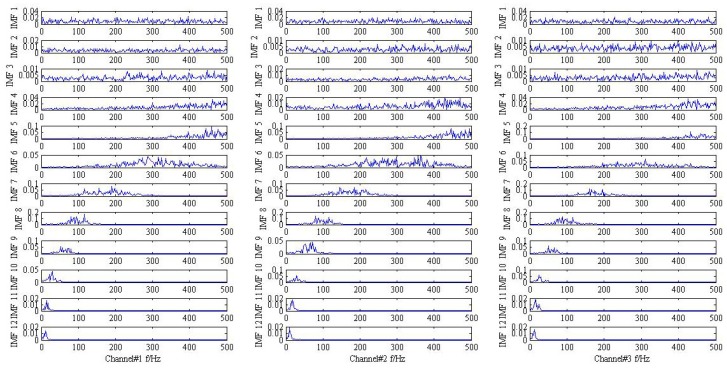
The result provided by MEMD in frequency domain.

The spectral analysis by FFT is applied to the measured trivariate signal in Figure 9 and the result shows that the signal components are complex. In addition, the fault characteristic frequency and frequency modulation phenomenon cannot be identified. Then, the conventional MEMD method is used to analysis the fault signal and the result in frequency-domain is plotted in Figure 10. It is also noted that all the analyses employed 32 projection vectors to capture the direction of highest curvature of trivariate signals. By observing the frequency features of each IMFs, the fault characteristic frequency still cannot be inspected.

**Figure 11:**
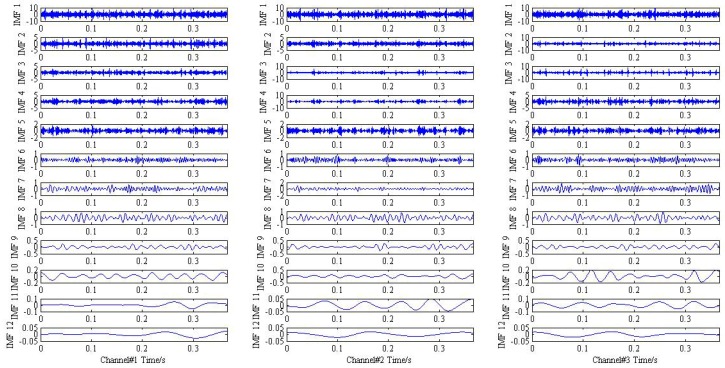
The result provided by the proposed method in time domain.

**Figure 12:**
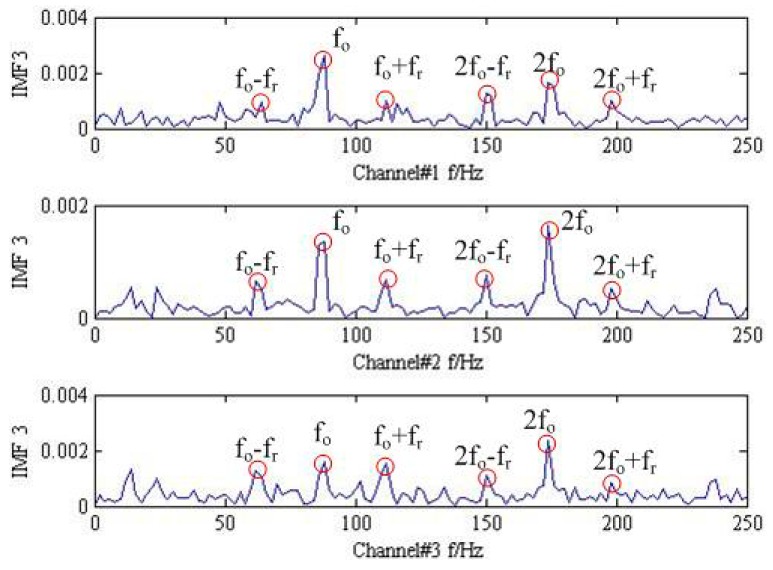
The frequency analysis result about the IMF3 by the proposed method.

**Figure 13:**
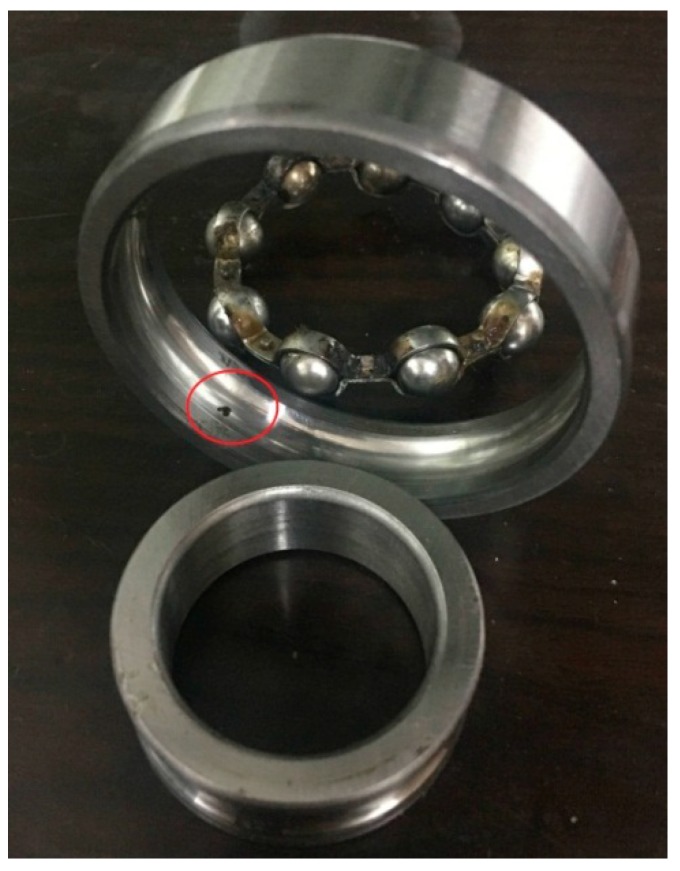
The picture of faulty bearing located in outer ring.

Finally, the proposed method is employed to the measured trivariate vibration signal and the result in time-domain is shown in Figure 11. For the sake of clarity, the FFT operators to the IMF3 for all the channels are conducted, and the corresponding results are plotted in Figure 12. Fortunately, the outer ring fault frequency $f_{o}$ and its doubling $2f_{o}$ can be found. Additionally, the special frequency modulation phenomenon of fault rolling bearing such as $f_{o}\pm f_{r}$ and $2f_{o}\pm f_{r}$ can also be identified. Thus, we can firmly believe that the fault is located on the outer ring, which is consistent with the actual situation shown in Figure 13.

By comparing the results respectively provided by MEMD and the method proposed in this paper, we can make a conclusion that the proposed method has better performance in trivariate signal mode decomposition and fault feature extraction.

## 5. Conclusions

Based on the traditional MEMD method, a novel method of trivariate empirical mode decomposition via convex optimization for rolling bearing condition identification is proposed and tested in this paper. The main research works are listed as follows: (1) a low-rank matrix approximation via convex optimization framework is proposed for the trivariate signal denoising. By introducing parameterized non-convex function, its performance is improved; (2) the principal component obtained by SVD operator is employed to indicate the sample directions, thus the non-uniform sample scheme is presented to meet the requirement of inhomogeneity of the data distribution in high-dimensional space; (3) through the analysis of the simulation signal and the measured vibration signal, it is demonstrated that the proposed method is superior to MEMD in multi-scale feature extraction. It can be regarded as powerful tool in trivariate signal processing.

## Figures and Tables

**Table 1:** The experimental parameters and fault frequency.

Rotating Speed r/min	Rotating Frequency/Hz	Sampling Frequency/Hz	Sampling Time/s	Outer Fault Frequency/Hz
1450	24.17	16,000	0.3661	87.01
